# Asteraceae Plants as Sources of Compounds Against Leishmaniasis and Chagas Disease

**DOI:** 10.3389/fphar.2019.00477

**Published:** 2019-05-08

**Authors:** Roberval Nascimento Moraes Neto, Ruth Flávia Barros Setúbal, Taciana Mirely Maciel Higino, Maria Carolina Accioly Brelaz-de-Castro, Luís Cláudio Nascimento da Silva, Amanda Silva dos Santos Aliança

**Affiliations:** ^1^Programa de Pós-Graduação da Universidade Ceuma, São Luís, Brazil; ^2^Departamento de Investigação Científica, Fundação Altino Ventura, Recife, Brazil; ^3^Departamento de Enfermagem, Universidade Federal de Pernambuco, Vitória de Santo Antão, Brazil

**Keywords:** asteraceae plants, trypanosomatids, *Trypanosoma cruzi*, trypanocidal activity, *Leishmania* spp., leishmanicidal activity

## Abstract

Leishmaniasis and Chagas disease cause great impact on social and economic aspects of people living in developing countries. The treatments for these diseases are based on the same regimen for over 40 years, thus, there is an urgent need for the development of new drugs. In this scenario, Asteraceae plants (a family widely used in folk medicine worldwide) are emerging as an interesting source for new trypanocidal and leishmanicidal compounds. Herein, we provide a non-exhaustive review about the activity of plant-derived products from Asteraceae with inhibitory action toward *Leishmania* spp. and *T. cruzi*. Special attention was given to those studies aiming the isolation (or identification) of the bioactive compounds. Ferulic acid, rosmarinic acid, and ursolic acid (*Baccharis uncinella* DC.) were efficient to treat experimental leishmaniasis; while deoxymikanolide (*Mikania micrantha*) and (+)-15-hydroxy-labd-7-en-17-al (*Aristeguietia glutinosa* Lam.) showed *in vivo* anti-*T. cruzi* action. It is also important to highlight that several plant-derived products (compounds, essential oils) from *Artemisia* plants have shown high inhibitory potential against *Leishmania* spp., such as artemisinin and its derivatives. In summary, these compounds may help the development of new effective agents against these neglected diseases.

## Introduction

Protozoa are unicellular eukaryotes that cause some of the most common diseases in humans and domestic animals. These parasites have a range of habitats within their hosts, living in various parts of the body during their life cycle (Ullah et al., 2017). The Trypanosomatidae family includes several human-infective protozoans, such as *Leishmania* spp., and *Trypanosoma cruzi*, and they cause Leishmaniasis and Chagas disease, respectively. They affect mainly people living in developing countries, causing great disruption in their quality of life. These diseases are considered neglected diseases by the World Health Organization (WHO, 2013).

Leishmaniasis is considered one of the most significant neglected tropical diseases (Feasey et al., 2010). It is endemic in 98 countries with 350 million people at risk of getting the disease. The mortality rate is 70.000 cases/per year worldwide. Leishmaniasis has an incidence of 0.5 million cases of the visceral form and 1.5–2.0 million cases of cutaneous form (Blum et al., 2004; Reithinger et al., 2007a,b; WHO, 2016). Currently, therapeutic approaches for controlling leishmaniasis comprises only five drugs: the pentavalent antimonial, amphotericin B and its liposomal formulation AmBisome, miltefosine, paromomycin, and pentamidine. These drugs are associated with serious problems such as toxicity and emergence of drug-resistant strains (Tiwari and Dubey, 2018; Tiwari et al., 2018).

Chagas disease (or American trypanosomiasis) is the main cause of heart failure by an infection in Latin America, where the morbidity and mortality associated with this disease is superior to other neglected ones (malaria, schistosomiasis, and leishmaniasis; Martins-Melo et al., 2016). About 10 million infection cases and 14.000 deaths are recorded per year (Coura, 2015). Benznidazole (BNZ), which was developed over 40 years ago, is the first-line drug for the treatment of Chagas disease (Davanço et al., 2016). BNZ shows good efficacy in the acute phase of the disease (80–90% cure), however its greatest restriction is the limited cure efficacy in the chronic phase, which is considered far of the ideal (8–20%) (Bern, 2015). In addition, treatment with BNZ presents other problems, such as high administered doses, long term treatment and high incidence of adverse reactions, which are probably related to the generation of reactive metabolites produced from the metabolism of BNZ (Palmeiro-Roldan et al., 2014; Bermudez et al., 2016).

Since pharmaceutical companies neglect these diseases, there is an urgent demand to accelerate the development of more effective drugs against them. Plants are emerging as interesting sources of new trypanocidal and leishmanicidal compounds. They hold the promise for improvements in the field of drug development, and the ethnomedicinal knowledge plays an essential role in this process (Bermudez et al., 2016). For example, several plants from the Asteraceae family have provided some lead molecules against *Leishmania* spp. and *T. cruzi* (Sülsen et al., 2008; Beer et al., 2016; García et al., 2017; Kimani et al., 2017; Laurella et al., 2017). Indeed, Asteraceae plants play important ethnopharmacological role worldwide making them attractive candidates for drug development (Ali et al., 2017; Carvalho et al., 2018; Fattori et al., 2018; Naß and Efferth, 2018).

**Figure 1:**
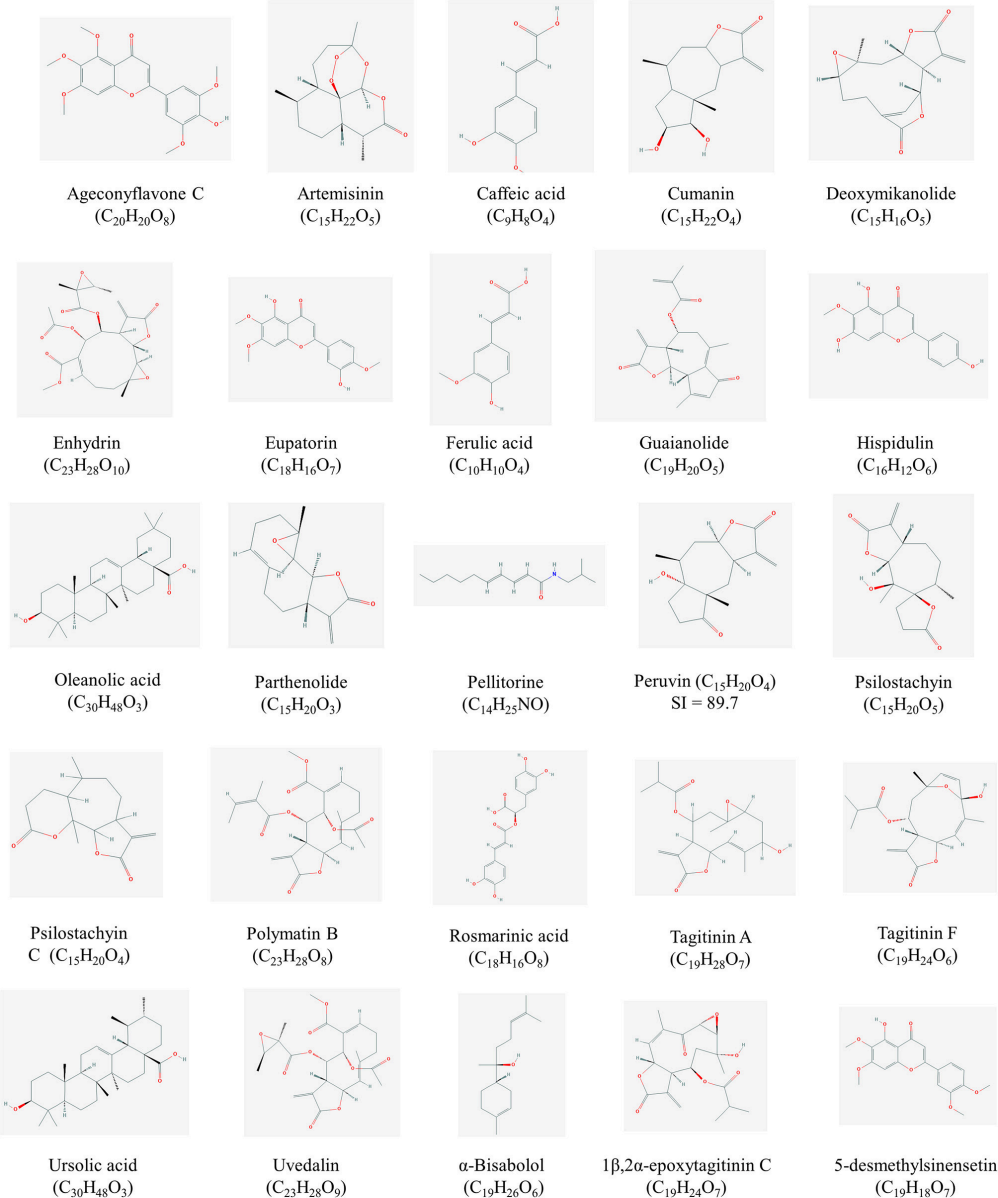
Structures of high promising selected compounds with activity against *Leishmania* spp. All structures were obtained from Pubchem (https://pubchem.ncbi.nlm.nih.gov/).

**Figure 2:**
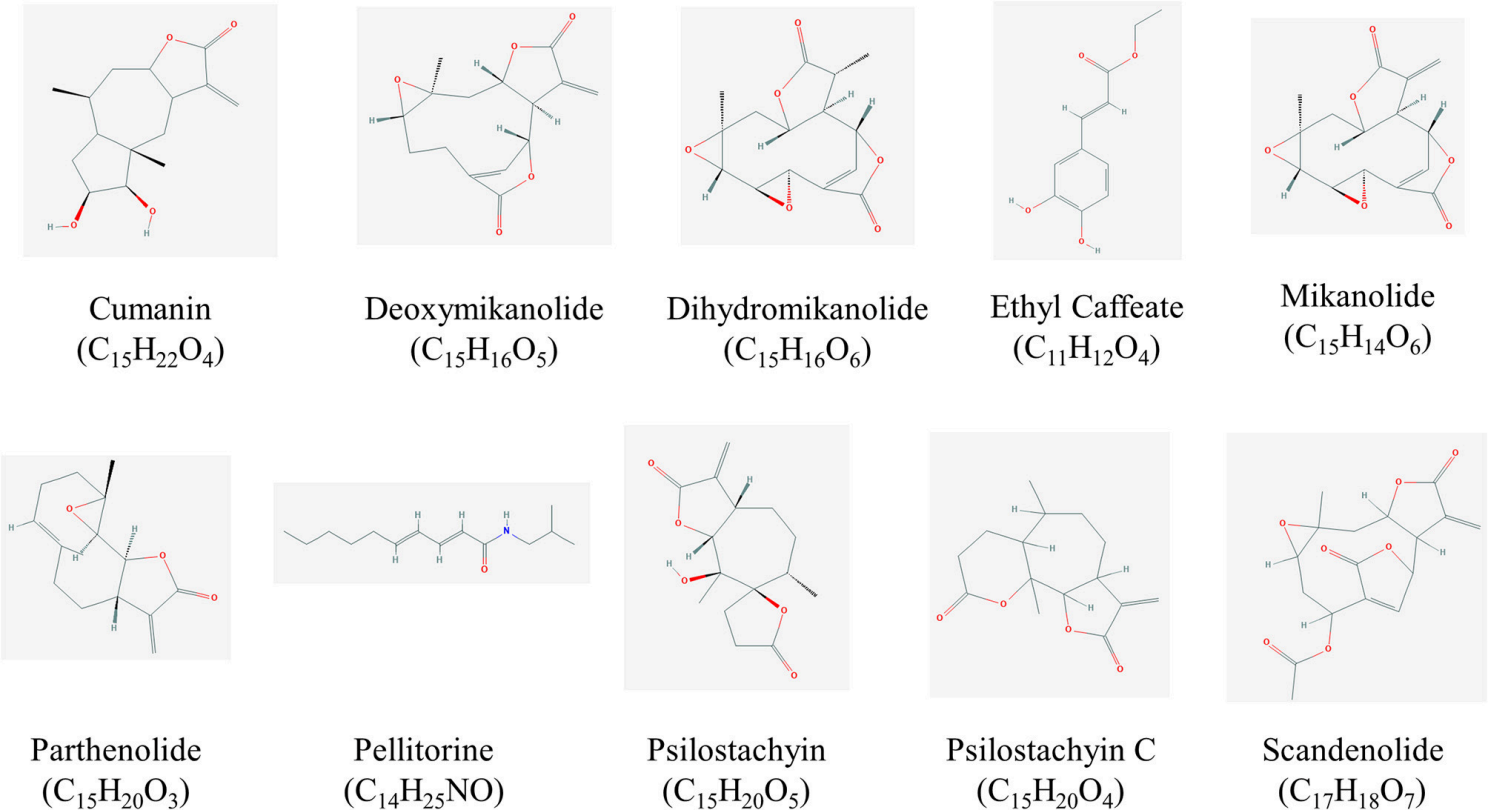
Structures of high promising selected compounds with activity against *T. cruzi*. All structures were obtained from Pubchem (https://pubchem.ncbi.nlm.nih.gov/).

This paper provides a non-exhaustive overview on the contribution of Asteraceae family for the development of leishmanicidal and trypanocidal drugs. The search for papers was done between January and December of 2018, in PUBMED and Google Scholar databases. Special emphasis was given to those studies about the isolation of bioactive compounds and/or their *in vivo* evaluation. The ethnomedicinal uses of the plants listed in this work are summarized in Table 1. In addition, the structures of the most promising compounds (those that presented Selective index ≥ 5) that are available at PubChem (https://pubchem.ncbi.nlm.nih.gov/) are shown in Figures 1, 2.

**Table 1:** Overview of selected Asteraceae plants with inhibitory activity toward Trypanosomatids.

**Plants**	**Ethnopharmacological relevance**	**Compounds with activity toward Trypanosomatids**	***Trypanosoma cruzi***	***Leishmania* sp**	**References**
*Achillea ptarmica*	Treatment of stomach and digestive disorders.	Pellitorine	8.45 ± 1.08 μg/mL^a^	5.96 ± 0.16 μg/mL^a, Ldo^	Rigat et al., 2009; Althaus et al., 2014
		8,9-Z-Dehydropellitorine	14.2 ± 2.5 μg/mL^a^	5.01 ± 0.12 μg/mL^a, Ldo^	
*Ageratum conyzoides*	Treatment of sleeping sickness, bleeding, leprosy, infectious diseases, headaches, allergies, skin diseases and dyspnea.	Ageconyflavone C	>30 μg/mL^a^	3.56 μg/mL^a, Ldo^	Okunade, 2002; Nour et al., 2010; Sharma et al., 2014
		5,6,7,5′-tetramethoxy-3′,4′-methylenedioxyflavone	19.5 μg/mL^a^	>30 μg/mL^a, Ldo^	
		Eupalestin	>30 μg/mL^a^	>30 μg/mL^a, Ldo^	
		5′-methoxynobiletine	26.4 μg/mL^a^	5.29 μg/mL^a, Ldo^	
		5,6,7,3′,4′,5′-hexamethoxyflavone	>30 μg/mL^a^	8.61 μg/mL^a, Ldo^	
*Aldama discolor*	-	Ent-3-α-hydroxy-kaur-16-en-18-ol	55.6 μM^a^	2.5 ± 1.5 μM^a, Ldo^	Nogueira et al., 2016
		Ent-7-oxo-pimara-8,15-diene-18-ol	15.4 μM^a^	18.2 μM^a, Ldo^	
		Ent-2S,4S-2-19-epoxy-pimara-8(3),15-diene-7β-ol	19.4 μM^a^	13.8 μM^a, Ldo^	
		Ent-7-oxo-pimara-8,15-diene-3β-ol	58.9 μM^a^	21.9 μM^a, Ldo^	
*Ambrosia elatior*	Used as contraceptive, antiprotozoal and expectorant agent and for the treatment of headache.	Cumanin	8 μM^a^	3 μM^p, Lam^	Sülsen et al., 2013; González et al., 2018
			180 μM^t^, ^#^/170 μM^t^, ^*^	19 μM^p^, ^Lam^	
			12 μM^e^, ^#^/4 μM^e^, ^*^	< 1 μM^p^, ^Lbr^	
*Ambrosia scabra*	Treatment of headache, rheumatism, pain and fever.	Psilostachyin	21 μM^a^	10 μM^p, Lam^	Gómez-Estrada et al., 2011; Sülsen et al., 2013; Alonso-Castro et al., 2017
				< 1 μM^p^, ^Lbr^	
		Cordilin	90 μM^b^, ^#^/83 μM^b^, ^*^	55 μM^p, Lam^	
			26 μM^d^, ^#^/44 μM^d^, ^*^	< 1 μM^p^, ^Lbr^	
		Daucosterol	> 174 μM^e^	NT	
	Used as anthelminthic and antipyretic agent.	Psilostachyin C	0.9 μg/mL^a^	1.2 μg/mL^p, Lme^	Sülsen et al., 2011, 2016
			3.5 μg/mL^t^	1.5 μg/mL^p, Lam^	
			0.6 μg/mL ^e^		
*Ambrosia tenuifolia*	Used as a carminative, anthelminthic and antipyretic agent.	Hispidulin	62.3 μM^t^	6.0 μM^p, Lme^	Sülsen et al., 2007, 2008
			46.7 μM^e^		
		Psilostachyin	0.76 μg/mL^t^	0.12 μg/mL^p, Lme^	
			1.22 μg/mL^e^		
		Peruvin	52.8 μg/mL^t^	0.39 μg/mL^p, Lme^	
			1.65 μg/mL^e^		
*Anacyclus pyrethrum*	Used as brain tonic and to treat inflammatory and painful diseases.	Undeca-2E, 4E-dien-8,10-diynoic acid isopentylamide	16.3 ± 0.3 μg/mL^a^	4.04 ± 0.71 μg/mL^a, Ldo^	Pahuja et al., 2012; Althaus et al., 2017; Manouze et al., 2017
		Tetradeca-2E,4E,12Z-trien-8,10-diynoic acid isobutylamide	38.8 ± 2.1 μg/mL^a^	5.04 ± 1.17 μg/mL^a, Ldo^	
		Deca-2E,4E,9-trienoic acid isobutylamide	39.9 μg/mL^a^	4.77 ± 1.02 μg/mL^a, Ldo^	
*Anthemis nobilis*	Used to treat infections, diabetes and ophthalmological, neurological and mental disorders.	Furanoheliangolide	37.3 ± 3.5 μM^a^	9.8 ± 0.2 μM^a, Ldo^	Calvo and Cavero, 2015, 2016; De Mieri et al., 2017
		8-tigloylhydroxyisonobilin	26.7 ± 0.2 μM^a^	5.3 ± 0.3 μM^a, Ldo^	
		Hydroxyisonobilin	29.3 ± 2.6 μM^a^	13.2 ± 0.07 μM^a, Ldo^	
		8-methacrylate nobilin	4.2 ± 0.5 μM^a^	NT	
		Seconobilin B	5.0 ± 0.6 μM^a^	0.38 ± 0.05 μM^a, Ldo^	
		Guaianonobilin	10.9 ± 0.4 μM^a^	0.8 ± 0.1 μM^a, Ldo^	
*Anthemis auriculata*	-	Anthecotulide	18.05 μg/mL^a^	8.18 μg/mL^a, Ldo^	Karioti et al., 2009
		4-hydroxyanthecotulide	5.72 μg/mL^a^	3.27 μg/mL^a, Ldo^	
		4-acetoxyanthecotulide	>30 μg/mL^a^	12.5 μg/mL^a, Ldo^	
*Aristeguietia glutinosa*	Treatment of rheumatism, ulcers, headaches, diarrhea and other infectious diseases.	(+)-15-hydroxy-labd-7-en-17-al	3.0 μg/mL^e^	NT	Varela et al., 2012, 2014
		(+)-13,14,15,16-tetranor-labd-7-en-17,12-olide	15.6 μg/mL^e^	NT	
*Artemisia annua*	Treatment of malaria.	Artemisinin	NT	22 μg/mL^a, Ldo^	Sen et al., 2007, 2010; Van Der Kooy and Sullivan, 2013
				160 μg/mL^p, Ldo^	
*Artemisia campestris*	Treatment of diabetes, gastric disorders, infections, hypertension and rheumatism.	None	NT	44 μg/mL^p, Lin^	Aloui et al., 2016; Pereira et al., 2018
*Artemisia herba-alba*	Treatment of diabetes, hypertension, spasmodic dysphonia and some bacterial infection.	None	NT	68 μg/mL^p, Lin^	Aloui et al., 2016;Laadraoui et al., 2018
*Baccharis retusa*	Treatment of several illnesses, including parasitic diseases.	5,6,7-trihydroxy-4′-methoxyflavanone	20.39 μg/mL^t^	45.39 μg/mL^a, Lch^	Grecco et al., 2010; Grecco Sdos et al., 2012; Ueno et al., 2018
				40.14 μg/mL^p, Lch^	
				53.95 μg/mL^p, Lam^	
				56.96 μg/mL^p, Lma^	
				49.71 μg/mL^p, Lbr^	
		Sakuranetin	20.17 g/mL^t^	43.66 μg/mL^a, Lch^	
				38.41 μg/mL^p, Lch^	
				51.89 μg/mL^p, Lam^	
				52.60 μg/mL^p, Lma^	
				45.12 μg/mL^p, Lbr^	
		ent-15β-senecioyl-oxy-kaur-16-en-19-oic acid	3.8 μmM^t^	NT	
		ent-kaur-16-en-19-oic acid	75.3 μM^t^	NT	
		ent-16-oxo-17-nor-kauran-19-oic acid	83.2 μM^a^	NT	
			44.2 μM^t^	NT	
*Baccharis uncinella*	Used as sedative agent.	Caffeic acid	51.61 μg/mL^t^	0.8 ± 0.5 ng/μL^a, Lam^	Passero et al., 2011; Jesus et al., 2017
				190 ± 70 ng/μL^p, Lam^	
		Pectolinaringenin	55.62 μg/mL^t^	60 ± 0.008 ng/μL^a, Lbr^	
				110 ± 30 μg/μL^p, Lbr^	
		Oleanolic acid	NT	20 ± 7.0 ng/μL^a, Lam^	
				210 ± 10 ng/μL^a, Lbr^	
		Ursolic acid	NT	410 ± 40 ng/μL^a, Lam^	
*Baccharis dracunculifolia*	Treatment of inflammatory disorders.	Isosakuranetin	247.6 ± 1.13 μM^t^	NA	Da Silva Filho et al., 2014, 2019
		Baccharis oxide	249.8 ± 1.02 μM^t^	NT	
		Aromadendrin-4'-methylether	947.7 ± 1.05 μM^t^	NA	
		Ferulic acid	1135.9 ± 1.07 μM^t^	NT	
		3-prenyl-4-(dihydrocinnamoyloxy)-cinnamic acid	523.8± 1.05 μM^t^	NT	
		Ursolic acid	NT	3.7 μg/mL^p,Ldo^	
		Hautriwaic acid lactone	NT	7.0 μg/mL^p,Ldo^	
*Calea uniflora*	Used as wound healing agent and to treat muscle pain.	2-senecioyl-4-(hydroxyethyl)-phenol	< 500 μg/mL^t^	NT	do Nascimento et al., 2004; Lima et al., 2016
		2-senecioyl-4-(pentadecanoyloxyethyl)-phenol	< 500 μg/mL^t^	NT	
*Mikania variifolia and Mikania micrantha*	Used as wound healing agent and as antidote against snake bites and scorpion sting.	Mikanolide	4.5 μg/mL^a^	5.1 μg/mL^p, Lbr^	Li et al., 2013; Laurella et al., 2017
			2.1 μg/mL^t^		
			0.7 μg/mL^e^		
		Deoxymikanolide	6.3 μg/mL^a^	11.5 μg/mL^p, Lbr^	
			1.5 μg/mL^t^		
			0.08 μg/mL^e^		
		Dihydromikanolide	8.5 μg/mL^a^	57.1 μg/mL^p, Lbr^	
			0.3 μg/mL^t^		
			2.5 μg/mL^e^		
*Pentacalia desiderabilis*	-	Jacarone	13 μg/mL^t^	17.22 μg/mL^p, Lch^	Morais et al., 2012
				12.93 μg/mL^p, Lbr^	
				11.86 μg/mL^p, Lam^	
*Porophyllum ruderale*	Used in folk medicine as leishmanicidal and anti-inflammatory agent.	5-methyl-2,2':5',2″-terthiophene	NT	19 μg/mL^a, Lam^	Takahashi et al., 2011
				7.7 μg/mL^p, Lam^	
		5'-methyl-[5-(4-acetoxy-1-butynyl)]-2,2'-bithiophene	NT	28.7 μg/mL^a, Lam^	
				21.3 μg/mL^p, Lam^	
*Pluchea carolinensis*	Treatment of migraine.	Caffeic acid	NT	2.9 ± 0.3 μg/mL^a, Lam^	Montrieux et al., 2014; García et al., 2017
				0.9 ± 02 μg/mL^c, Lam^	
		Chlorogenic acid	NT	1.9 ± 0.5 μg/mL^a, Lam^	
				0.2 ± 0.05 μg/mL^p, Lam^	
		Ferulic acid	NT	1.5 ± 0.1 μg/mL^a, Lam^	
				0.3 ± 0.3 μg/mL^p, Lam^	
		Quercetin	NT	1.3 ± 0.1 μg/mL^a, Lam^	
				0.2 ± 0.06 μg/mL^p, Lam^	
		Rosmarinic acid	NT	1.7 ± 0.4 μg/mL^p, Lam^	
				0.2 ± 0.1 μg/mL^p, Lam^	
*Stevia satureifolia*	–	Eupatorin	NA	55.1 μg/mL^p, Lbr^	Beer et al., 2016
			0.2 μg/mL^e^		
			61.8 μg/mL^t^		
		5-desmethylsinensetin	78.8 μg/mL^a^	37 μg/mL^p, Lbr^	
			0.4 μg/mL^e^		
			75.1 μg/mL^t^		
*Tithonia diversifolia*	Treatment of wounds, diabetes, skeleto-muscular disorders, abscesses, dermatological conditions, and stomach pains, as well as malaria, fever, hepatitis and other infectious diseases.	1β, 2α-epoxytagitinin C	NT	2.2 ± 0.9 μg/mL^p, Lbr^	De Toledo et al., 2014; Mabou Tagne et al., 2018
		Tagitinin F	NT	7.4 ± 2.8 μg/mL^p, Lbr^	
		Tagitinin A	NT	7.5 ± 3.2 μg/mL^p, Lbr^	
		Guaianolide 7	NT	9 ± 1.2 μg/mL^p, Lbr^	
		Tirotundin 3-O-methyl ether	NT	13.7 ± 2.6 μg/mL^p, Lbr^	
		Tirotundin	NT	8.7 ± 1.9 μg/mL^p, Lbr^	
		Tagitinin C	NT	3.2 ± 0.5 μg/mL^p, Lbr^	
*Vernonia polyanthes*	Treatment of skin diseases, inflammation, rheumatism, as well as a healing agent.	Zerumbone	NT	9 μg/mL^p, Lin^	Rodrigues et al., 2016; Moreira et al., 2017
*Vernonia scorpioides*	Treatment of skin diseases, including skin parasites, allergies, irritations, itching and chronic wounds.	Lup-20(29)-ene-diol	12.4 μg/mL^a^	ND	Machado et al., 2018

a, amastigote; t, trypomastigote; p, promastigote; e, epimastigote; Lam, L. amazonenses; Lbr, L. braziliensis; Lch, L. chagasi; Ldo, L. donovani; Lin, L. infantum; Lme, L. mexicana; NT, Not tested; ND, Not detected; NA, Not active;

# T. cruzi RA strain;

* *T. cruzi K98 strain*.

## Pellitorine and 8,9-Z-Dehydropellitorine From *Achillea ptarmica* L. Are Active Against Trypanosomatids

Extracts and isolated compounds of *Achillea ptarmica* L. flowers were tested against amastigote forms of *L. donovani* and *T. cruzi*. The cytotoxicity effects of each sample was evaluated using L6 cells (rat skeletal myoblasts), revealing that Pellitorine and 8,9-Z-Dehydropellitorine were the most promising compounds toward *L. donovani* [Selectivity Index (SI) of 7.6 and 3.2, respectively]. Pellitorine also showed activity against *T. cruzi* (SI: 5.34) (Althaus et al., 2014).

## Compounds From *Ageratum conyzoides* L. Are Active Against Trypanosomatids

*Ageratum conyzoides* L. is another Asteraceae plant with activity against trypanosomatids. The dichloromethane extract from *A. conyzoides* aerial parts was found to inhibit *L. donovani* [IC50 (concentration that inhibit 50% of parasite growth) = 3.4 μg/mL] and *T. cruzi* (IC50 = 29 μg/mL). The bio-guided fractionation allowed the isolation of the active compounds. Ageconyflavone C had the highest activity against *L. donovani* (IC50 = 3.56 μg/mL) with no measurable toxicity against L6 cells (SI >25.28); followed by 5′-methoxynobiletine (SI = 3.6). 5,6,7,5′-Tetramethoxy-3′,4′-methylenedioxyflavone was the most active compound against *T. cruzi* (IC50 = 19.5 μg/mL; SI >4.6; Nour et al., 2010). Encecalol angelate is another compound detected in the freshly prepared dichloromethane extract of *A. conyzoides*. As encecalol angelate was found to be unstable, a synthetic approach was employed to obtain this compound; however it displayed low antiprotozoal activity (Harel et al., 2011).

In addition, other report showed that the hydroalcoholic extract of *A. conyzoides* aerial parts inhibited promastigotes and trypomastigotes forms of *L. amazonensis* and *T. cruzi* (IC50 values of 107 and 104.7 μg/mL, respectively), as well as the infective abilities of *L. amazonensis* and *T. cruzi* (Teixeira et al., 2014). However, the extract showed toxicity against J774.G8 macrophages.

## Diterpenoids From *Aldama discolor* Are Active Against Trypanosomatids

*Aldama discolor* (syn. *Viguiera discolor* Baker.) is an endemic plant from Brazilian Cerrado that showed inhibitory action against *L. donovani* and *T. cruzi*. Four diterpenoids were isolated from dichloromethane extract of *A. discolor* leaves: *ent*-3-α-hydroxy-kaur-16-en-18-ol, *ent*-7-oxo-pimara-8,15-diene-18-ol, *ent*-2S,4S-2-19-epoxy-pimara-8(3),15-diene-7β-ol and *ent*-7-oxo-pimara-8,15-diene-3β-ol. All compounds inhibited the growth of the amastigote forms from *L. donovani* and *T. cruzi*. Based on SI values obtained using L6 cells, the compound *ent*-3-α-hydroxy-kaur-16-en-18-ol (IC50 = 2.5 μM; SI = 16) showed the highest effect against *L. donovani*; while *ent*-7-oxo-pimara-8,15-diene-18-ol (IC50 = 15.4 μM; SI = 3) and *ent*-2S,4S-2-19-epoxy-pimara-8(3),15-diene-7β-ol (IC50 = 19.4 μM; SI = 4) were the most active against *T. cruzi* (Nogueira et al., 2016).

## Compounds From *Ambrosia* Plants Are Active Against Trypanosomatids

The trypanocidal activity of *Ambrosia* plants was reported (Sülsen et al., 2006) and some active compounds were isolated, such as hispidulin (Sülsen et al., 2007), psilostachyin, and peruvin from *Ambrosia tenuifolia* Spreng (Sülsen et al., 2008); cumanin from *Ambrosia elatior* L. (Sülsen et al., 2013); damsin and confertin from *Ambrosia peruviana* Willd. Aponte et al. (2010); psilostachyin, cordilin, daucosterol (Sülsen et al., 2013), and psilostachyin C (Sülsen et al., 2011) from *Ambrosia scabra* Hook. & Arn.

Hispidulin is a flavonoid isolated from the aerial parts of *A. tenuifolia* that showed action against epimastigotes (IC50 = 46.7 μM; SI> 3.6) and trypomastigotes (IC50 = 62.3 μM; SI> 2.7) forms of *T. cruzi*; and it was high activity against *L. mexicana* promastigotes (IC50 = 6.0 μM; SI > 27.8). The toxicity was evaluated using lymphoid cells (Sülsen et al., 2007). This compound was also isolated from the aerial parts of *Baccharis uncinella* showing action against *T. cruzi* (Grecco Sdos et al., 2014). Although hispidulin has shown promising activity against these trypanosomatids, there are no reports about its *in vivo* action.

Two sesquiterpene lactones were obtained from the aerial parts of *A. tenuifolia* (psilostachyin and peruvin) with anti-*T. cruzi* action (both with an IC50 of 2 μg/mL against epimastigotes forms). The authors also demonstrated the *in vivo* action of psilostachyin [the most active against trypomastigote forms; with an IC50 of 0.76 μg/mL and SI of 33.8 (tested using T lymphocytes)]. In addition, psilostachyin and peruvin also showed even higher activity against *L. mexicana* promastigotes with an IC50 values of 0.12 μg/mL (SI = 214.2) and 0.39 μg/mL (SI = 89.7), respectively. In the experimental model of Chagas disease, the treatment with psilostachyin (or benznidazole) started 5 days post-infection; and it was performed by intraperitoneal route for 5 days (1 mg/kg of body weight/day). All psilostachyin-treated animals survived, while the mice in the other groups (untreated mice or animals treated with benznidazole) died after 35 days (Sülsen et al., 2008). However, other study reported that psilostachyin was not efficient in an acute model of *T. cruzi* infection. These different results may be explained by the differences in the treatment schedule in each study (Da Silva et al., 2013).

The anti-*T. cruzi* activity of the sesquiterpene lactone psilostachyin C isolated from *A. scabra* was also reported (Sülsen et al., 2011). In this study, the authors showed that psilostachyin C inhibited all forms of *T. cruzi* with low IC50 values (epimastigotes: 0.6 μg/mL; trypomastigotes: 3.5 μg/mL; and amastigotes: 0.9 μg/mL) and high SI values (145.83, 97.22, and 25, respectively; when tested against murine peritoneal macrophages). The action of psilostachyin C on *T. cruzi* epimastigotes was associated with the induction of multivesicular bodies and vacuolization. Moreover, psilostachyin C also showed *in vitro* activity against the promastigote forms of *L. mexicana* (IC50 = 1.2 μg/mL; SI = 72.92) and *L. amazonensis* (IC50 = 1.5 μg/mL; SI = 58.33). Due the higher anti-*T. cruzi* properties of psilostachyin C, the *in vivo* effects were evaluated in a murine model of Chagas disease. The administration of psilostachyin C (1 mg/kg/day during 5 days) to animals with 5 days of *T. cruzi* infection resulted in the reduction of parasitaemia and increased survival, a result similar to benznidazole (Sülsen et al., 2011).

Later, the mechanisms involved in the anti-*T. cruzi* actions of both psilostachyin (from *A. tenuifolia*) and psilostachyin C (from *A. scabra*) were evaluated by a range of *in vitro* assays. The study revealed that despite their chemical similarities and the fact that both compounds activated the apoptosis pathways, the effects of each compound are associated with different targets on epimastigotes forms: psilostachyin interact with hemin and psilostachyin C with sterol synthesis. In addition, the treatment with psilostachyin resulted in a 5-fold increase in the levels of reactive oxygen species (ROS), while psilostachyin C lead to a 1.5 increase in ROS quantities (Sülsen et al., 2016). These results may be associated to the ultrastructural alterations induced by psilostachyin that included mitochondrial swelling and kinetoplast abnormality (Sülsen et al., 2010). These effects were observed to psilostachyin C-treated parasites (Sülsen et al., 2011).

Other compound from the *Ambrosia* plants with promising action against trypanosomatids is cumanin, a sesquiterpene lactone isolated from *A. elatior*. Cumanin showed leishmanicidal (IC50 of 19 μM against promastigote forms of *L. braziliensis* and *L. amazonensis*) and anti-*T. cruzi* activities (IC50 of 8, 12, and 180 μM against amastigote, epimastigote and trypomastigote forms, respectively). The *in vivo* action of cumanin was also demonstrated in an experimental model of Chagas disease induced by intraperitoneal injection of the RA strain. Cumanin was administrated (1 mg/kg of body weight/day by intraperitoneal route) for 5 days after the 5th day of parasite infection. The treatment with cumanin resulted in the survival of the *T. cruzi-*infected mice and in the reduction of parasitemia, effects similar to those found in the treatment with benznidazole. Moreover, this work also highlighted that cordilin was also active against *T. cruzi* (epimastigotes and trypomastigotes; Sülsen et al., 2013).

## Alkamides From *Anacyclus pyrethrum* (L.) Link Are Active Against *L. donavani*

The dichloromethane extract obtained from the roots of *Anacyclus pyrethrum* (L.) Link was used for the isolation of alkamides with activity against *L. donavani*. L6 cells were used to evaluate the toxicity of each compound. Among the alkamides, undeca-2*E*,4*E*-dien-8,10-diynoic acid isopentylamide showed the best activity (SI = 7), followed by tetradeca-2*E*,4*E*,12Z-trien-8,10-diynoic acid isobutylamide (SI = 3.9) and deca-2*E*,4*E*,9-trienoic acid isobutylamide (SI = 3.5) (Althaus et al., 2017).

## Compounds Derived From *Anthemis* Plants Are Active Against Trypanosomatids

*Anthemis nobilis* L. [synonym of *Chamaemelum nobile* (L.) All.] is a plant know as Roman chamomile and used in folk medicine to treat infections and other disorders (Calvo and Cavero, 2015, 2016). The dichlomethane extract prepared from flowers of *A. nobilis* potently inhibited *L. donovani* promatigote forms (IC50 = 1.40 μg/mL). Several compounds were isolated from this extract and their activity was evaluated against *T. cruzi* intracellular amastigotes and *L. donovani* axenically grown amastigotes; while their toxicity was evaluated against L6 cells. Regarding the action against *T. cruzi*, the most active compound was 8-methacrylate nobilin (IC50 = 4.2 μM; SI = 6.1). Finally, seconobilin B (IC50 = 0.5 μM; SI = 11.2) and guaianonobilin (IC50 = 0.8 μM; SI = 7.2) showed the highest action against *L. donovani* (De Mieri et al., 2017). The aerial parts of *Anthemis auriculata* Boiss. have been also shown as sources of sesquiterpene lactones with anti-protozoa compounds: anthecotulide, 4-hydroxyanthecotulide, and 4-acetoxyanthecotulide. However, these compounds showed toxicity against L6 cells (Karioti et al., 2009).

## Compounds Isolated From *Aristeguietia glutinosa* Are Active Against *T. cruzi*

The hydroalcoholic extract from aerial parts of *Aristeguietia glutinosa* (Lam.) R.M.King & H.Rob and two diterpenoids [(+)-15-hydroxy-labd-7-en-17-al and (+)-13,14,15,16-tetranor-labd-7-en-17,12-olide] showed anti-*T. cruzi* action with IC50 values of 19.3, 3.0, and 15.6 μg/mL, respectively. The compounds also showed low toxicity toward erythrocytes and murine macrophages (Varela et al., 2012). The *in vivo* actions of the hydroalcoholic extract (50 mg/kg) and (+)-15-hydroxy-labd-7-en-17-al (10 mg/kg or 30 mg/kg) were evaluated in a BALB/c mice model of Chagas disease, and the treatment started 7 days after the infection. These agents reduced the amount of parasite in the blood leading to an increase in animal survival rates (Varela et al., 2014).

## Compounds Isolated From *Artemisia* Plants Are Active against *Leishmania* spp.

The genus *Artemisia* is composed by plants used for different ethnomedicinal practices (Bora and Sharma, 2011; Olennikov et al., 2018) and some *Artemisia*-derived compounds are promising anti-protozoa agents (Emami et al., 2012). In addition, a recent review showed the application of *Artemisia* plants and their constituents against Trypanosomiasis (Naß and Efferth, 2018). Since several papers evaluated the leishmanicidal effects of the *Artemisia* genus, in this section we reviewed studies where *in vivo* assays were employed along with the identification of the active(s) compound(s). In this sense, besides the studies discussed in this section, anti-*Leishmania* properties were also reported for extracts of *Artemisia absinthium* L. (Azizi et al., 2016), *Artemisia dracunculus* L. (Mirzaei et al., 2016; Rezaei et al., 2017), and *Artemisia seiberi* L. (Esavand Heydari et al., 2013).

Essential oils (EO) from some *Artemisia* plants have been pointed as interesting leishmanicidal agents (Abad et al., 2012), such as those obtained from *Artemisia ludoviciana* Nutt. (Baldemir et al., 2018) and *Artemisia abyssinica* Sch.Bip. ex A.Rich. (Tariku et al., 2010). For some of them, the *in vivo* properties were demonstrated; as an example the EO from *Artemisia absinthium* L. has inhibitory effects toward *L. amazonensis* (Monzote et al., 2014). *A. absinthium* EO was also evaluated against *L. amazonensis* in a murine model of experimental cutaneous leishmaniasis. The treatment with this oil (30 mg/kg by intralesional route) was able to reduce the lesion size and parasite burden, even when compared with mice treated with glucantime (Monzote et al., 2014).

The EO from *A. absinthium* was also reported as active against *L. aethiopica* and *L. donovani* (Tariku et al., 2011). All these good results lead to the development of a new formulation of *A. absinthium* EO using nanocochleates. Although the formulation exhibited lower efficacy against the amastigote form of *L. amazonensis*, the animals that received 4 administrations with this nanoformulation (30 mg/kg by intralesional route) for 4 days exhibited smaller lesion size than the untreated mice or those treated with EO itself. The results were similar to those obtained with Glucantime® treatment (Tamargo et al., 2017).

The EOs from *Artemisia campestris* (L.) and *Artemisia herba-alba* (Asso.) were tested against promastigote forms of *L. infantum* showing IC50 values of 44 and 68 μg/mL, respectively. The CC50 values obtained on peritoneal macrophages from BALB/c treated with *A. campestris* and *A. herba-alba* were 124.4 and 160 μg/mL, respectively, corresponding to a SI value of 2.82 for *A. campestris* and 2.35 for *A. herba-alba*. These oils showed different chemical compositions: *A. campestris* EO was mostly composed by monoterpene hydrocarbons (87%) and its major compound was β-pinene (32.95%); while *A. herba-alba* had high content of oxygenated monoterpenes (85.79%) and its major compound was camphor (36.82%). However, besides these chemical differences, the mechanisms of action of both EOs were related to apoptosis induction and cell cycle arrest (Aloui et al., 2016).

The EO obtained from leaves of *Artemisia annua* Pall. has also be shown as a potential alternative agent against Leishmaniasis. This EO has IC50 values of 14.63 μg/mL against promastigotes and 7.3 μg/mL against *L. donovani* amastigotes, without provoking toxic effects in RAW 264.7 macrophages (when tested up to 200 μg/mL). This EO induced parasite apoptosis and its intra-peritoneal administration (200 mg/kg) was effective in the treatment of experimental *L. donovani-*infected BALB/c mice. The major compounds of this oil were camphor (52.06%) and β-caryophyllene (10.95%) (Islamuddin et al., 2014).

Another report showed that n-hexane fraction from leaves and seeds of *A. annua* were active against *L. donovani* promastigotes (IC50 of 14.4 and 14.615 μg/mL, respectively) and amastigotes forms (IC50 of 6.6 and 5.05 μg/mL, respectively) and these effects were also related to apoptosis induction. The major compounds found in the leaves hexanic fraction were α-amyrinyl acetate and β-amyrine; while the seed fraction showed cetin and nonacosane (EINECS 211-126-2). Both fractions were composed by derivatives of artemisinin (Islamuddin et al., 2012).

Artemisinin is a sesquiterpene lactone isolated from *A. annua*. Artemisinin and its derivatives were shown to inhibit *L. donovani, L. infantum*, and *L. major* (through the induction of parasite apoptosis; Sen et al., 2007, 2010; Cortes et al., 2015; Ghaffarifar et al., 2015). Due its lipophilic character, some leishmanicidal formulations containing artemisinin were already evaluated in models *in vitro* and *in vivo*, as examples: poly lactic co-glycolic acid nanoparticles (Want et al., 2014, 2015, 2017) and nanoliposomes (Want et al., 2017).

Later, it was demonstrated the *in vivo* action of the n-hexane fractions from leaves and seeds of *A. annua* in a murine model of visceral leishmaniasis caused by *L. donovani*. The authors reported that besides inducing direct inhibition of parasite growth, these extracts also activated the Th1 response with generation of immunological memory (Islamuddin et al., 2015). The efficacy of *A. annua* powder leaves was also confirmed in humans, where patients received capsules containing its powder (total of 30 g) for over 20 days. Although this study only evaluated two patients, it is important to highlight that both were healed after the treatment and without any adverse effects or manifestations of the disease even up to 24 months after the cure (Mesa et al., 2017).

## Compounds From *Baccharis* Genus Are Active Against Trypanosomatids

In relation to the *Baccharis* genus, three species have been reported as promising candidates for drug development: *Baccharis retusa* DC., *B. uncinella* DC., *Baccharis dracunculifolia* DC. The methanolic extracts from *B. retusa* leaves showed action against *Leishmania* spp. and *T. cruzi*, and a flavonoid (5,6,7-trihydroxy-4′-methoxyflavanone) was isolated. This compound inhibited both parasites, being better against *T. cruzi* trypomastigotes (IC50 = 20.39 μg/mL), however it showed moderate toxicity toward THP-1 (SI = 2.43) and MK2 (SI = 0.66) cells (Grecco et al., 2010). Sakuranetin is another flavonoid extracted from *B. retusa* that showed activity against *T. cruzi* trypomastigotes (IC50 = 20.17 μg/mL) and *Leishmania* spp. promastigotes (IC50 = 43 μg/mL to 52 μg/mL), however it also showed significant toxicity on peritoneal macrophages from BALB/c mice (Grecco Sdos et al., 2012).

A recent work reported the isolation of three diterpenes from the aerial parts of *B. retusa* with anti-*T. cruzi* properties: e*nt*-15β-senecioyl-oxy-kaur-16-en-19-oic acid; *ent*-kaur-16-en-19-oic acid; and *ent*-16-oxo-17-nor-kauran-19-oic acid. These compounds were more effective against trypomastigotes, and only *ent*-16-oxo-17-nor-kauran-19-oic acid was active against the amastigote form. The most active against the trypomastigotes forms was e*nt*-15β-senecioyl-oxy-kaur-16-en-19-oic acid (IC50 = 3.8 μM; SI = 50 as determined using NCTC cells-clone L929) and its effects were related to interference in the permeability of the plasma membrane of the parasite (probably due its lipophilic characteristics; Ueno et al., 2018).

Anti-*Leishmania* compounds (caffeic acid, pectolinaringenin; and one fraction composed by oleanolic acid and ursolic acid) were isolated from ethanolic extract of *B. uncinella* aerial parts. These compounds exhibited low cytotoxicity toward J774 macrophages. Pectolinaringenin and the combination of oleanolic and ursolic acids were appointed as the most active compounds against amastigote forms of *L. amazonensis* and *L. braziliensis* (Passero et al., 2011). In a similar work, the action of the compounds isolated from ethanolic extracts of *B. uncinella* aerial parts were also analyzed against *T. cruzi*: caffeic acid (IC50 = 51.61 μg/mL), pectolinaringenin (IC50 = 55.62 μg/mL), hispidulin (IC50 = 80.61 μg/mL) and a mixture of three chrogenic acids (3,4-, 3,5-, and 4,5-O-dicaffeoylquinic acids; IC50 = 61.04 μg/mL) (Grecco Sdos et al., 2014).

These *in vitro* results encouraged the evaluation of the leishmanicidal properties of the fraction containing oleanolic and ursolic acids obtained from leaves of *B. uncinella* in a model of Tegumentar Leishmaniasis induced by *L. amazonensis*. Mice treated with this triterpenic fraction (at 1.0 or 5.0 mg/kg) showed lower levels of parasitism in the skin and decreased lesion size than untreated animals. These effects were similar to those observed for amphotericin B-treated mice. In both fraction-treated groups were also observed high amounts of interleukin-12 and interferon gamma (Yamamoto et al., 2014).

Later, it was reported that ursolic acid showed more potent action against *L. amazonensis* promastigotes than oleanolic acid. The effects of ursolic acid toward promastigotes were associated with activation of programmed cell death in a pathway dependent of mitochondria activity but not related to caspase 3/7. Only ursolic acid was able to eradicate the amastigotes by increasing the release of nitric oxide by peritoneal macrophages. The efficacy of ursolic acid was also proven *in vivo* using BALB/c mice infected *L. amazonensis* (Yamamoto et al., 2015). However, oleanolic acid has been highlighted in other works as an important lead molecule for development of drugs for treatment of leishmaniosis (Sifaoui et al., 2014, 2017; Ghosh et al., 2016; Melo et al., 2016; Pertino et al., 2017).

Recently, the ursolic acid obtained from leaves of *B. uncinella* was also shown as a potent agent against experimental visceral leishmaniasis caused by *L. infantum*. The intraperitoneal injection of ursolic acid (1.0 or 2.0 mg/kg) reduced the parasites load in spleen and liver, induced the proliferation of splenic mononuclear cells and the production of IFN-γ and nitric oxide (Jesus et al., 2017). Additionally, a nanostructured lipid carrier system coated with N-octyl-chitosan surface for improve the delivery of ursolic acid was developed for treatment of visceral leishmaniosis induced by *L. donovani*. The oral treatment with this preparation was more effective than free ursolic acid treatment and reduced the parasite load in the spleen (Das et al., 2017).

Regarding *B. dracunculifolia* (the most important source of the Brazilian green propolis), the extract from leaves showed anti-*T. cruzi* effects and five active compounds were obtained; among them, isosakuranetin and baccharis oxide showed the best inhibitory potentials with IC50 values of 247.6 and 249.8 μM, respectively. Other compounds [aromadendrin-4'-methylether, ferulic acid, and 3-prenyl-4-(dihydrocinnamoyloxy)-cinnamic acid] were classified as moderated inhibitors. The authors did not evaluated the toxicity of these compounds (Da Silva Filho et al., 2014). On the other hand, the most active anti*-L. donovani* agents obtained from *B. dracunculifolia* were ursolic acid (IC50 = 3.7 μg/mL) and hautriwaic acid lactone (IC50 = 7.0 μg/mL; Da Silva Filho et al., 2014). Further, the EO from leaves of *B. dracunculifolia* showed action against the promastigote forms of *L. donovani* (IC50: 42 μM). This oil had (E)-nerolidol (33.51%) and spathulenol (16.24%) as major compounds. The oil was not toxic to Vero cells at the tested concentrations (Parreira et al., 2010).

## Compounds Isolated From *Calea* Plants Are Active Against Trypanosomatids

In relation to plants belonging to the *Calea* genus, anti-trypanosomatids compounds have been isolated from two species: *Calea pinnatifida* (R.Br.) Less. and *Calea uniflora* Less. This last species is a plant with ethnomedicinal importance in the state of Santa Catarina (Brazil), however there are few scientific studies about its pharmacological properties (Ramos et al., 2016). Two p-hydroxyacetophenone derivatives [2-senecioyl-4-(hydroxyethyl)-phenol and 2-senecioyl-4-(pentadecanoyloxyethyl)-phenol] obtained from dichloromethane extract of *C. uniflora* reduced the viability of *T. cruzi* trypomastigotes by 70 and 71%, respctively (at a 500 μg/mL dose) (do Nascimento et al., 2004). Similarly, two chromanones [uniflorol-A and uniflorol-B] from this extract inhibited 88.9% of *L. major* promastigotes growth at a concentration of 100 μg/mL (Do Nascimento et al., 2007). The authors did not report the toxicity of these compounds above discussed.

Other compounds with promising inhibitory action toward *T. cruzi* amastigotes were isolated from dichloromethane and ethyl acetate fractions of *C. uniflora* leaves. Among them, ethyl caffeate showed the best activity with an IC50 of 18.27 μg/mL (SI = 12.95), while the mixture of butein and orobol (1:1) showed an IC50 of 26.53 μg/mL (SI = 3.61). The toxicity of these compounds was evaluated using THP-1 cells. The author also investigated the inhibitory action of the compounds isolated from *C. uniflora* leaves against *L. amazonensis* amastigotes, however no promising results were found (Lima et al., 2016).

In another work, two chromenes extracted from leaves of *C. pinnatifida* showed moderate activity against *L. amazonensis* amastigotes: 6-acetyl-7-hydroxy-2,2-dimethylchromene (eupatoriochromene; inhibition of 39.3%) and 6-(1-Hydroxyethyl)-7-methoxy-2,2-dimethylchromene (encecalinol; inhibition of 32.3%). The authors only performed an inhibition assay using the dose of 50 μg/mL; and the toxicity of these compounds was not reported in this study (Lima et al., 2015). Later, the compound 11,13-dihydroxy-calaxin (a new furanoheliangolide sesquiterpene lactone) was able to inhibit amastigotes of *T. cruzi* and *L. amazonensis*, when tested at 50 μM, however, this compound showed high cytotoxicity against THP-1 cell (the SI was not determined; Lima et al., 2017).

## Sesquiterpenes Isolated From *Mikania* Species Are Active Against *T. Cruzi*

The genus *Mikania* has been pointed as a source of bioactive compounds, based on this, the extracts of four species (*Mikania micrantha* Kunth, *Mikania parodii* Cabrera*, Mikania periplocifolia* Hook. & Arn, and *Mikania cordifolia* (L.f.) Willd.) were evaluated against *T. cruzi* and *L. braziliensis*. The organic extracts (prepared with dichloromethane/methanol solution; 1:1) of the four *Mikania* species exhibited inhibitory activity against both pathogens, however the *M. micrantha* extract was the most active, inhibiting by 77.6 and 84.9% the growth of epimastigotes and promastigotes of *T. cruzi* and *L. braziliensis*, respectively (Laurella et al., 2012).

Later, sesquiterpene lactones with inhibitory action against *T. cruzi* and *L. braziliensis* were obtained from dichloromethane extracts of *M. micrantha* and *Mikania variifolia* Hieron. The obtained compounds inhibited the amastigote and trypomastigote stages of *T. cruzi*. The higher SI values (as determined using human monocyte leukemia THP1 cells) for trypomastigotes were found to deoxymikanolide (SI = 54) and dihydromikanolide (SI = 49.9), followed by scandenolide (SI = 12.6) and mikanolide (SI = 10.7); while for amastigotes the order was scandenolide (SI = 14.2), deoxymikanolide (SI = 12.5), mikanolide (SI = 4.3), and dihydromikanolide (SI = 1.5). Furthermore, mikanolide (IC50 = 5.1 μg/mL; SI = 4.4) and deoxymikanolide (IC50 = 11.5 μg/mL; SI = 6.9) also demonstrated strong inhibitory effects toward *L. braziliensis*. Based on SI index for both amastigote and trypomastigote forms, deoxymikanolide was also evaluated in an *in vivo* model of lethal *T. cruzi* infection, where it reduced the parasite load and increased the mice survival (Laurella et al., 2017). The anti-*T. cruzi* activity of deoxymikanolide is related to reduction of thiol groups leading to more susceptibility for oxidative stress, inhibition of parasite antioxidant defense and induction of mitochondrial dysfunction (Puente et al., 2018).

## Jacaranone From *Pentacalia desiderabilis* Is Active Against Trypanosomatids

Jacaranone is a compound extracted from leaves of *Pentacalia desiderabilis* (Vell.) Cuatrec that showed inhibitory action against *L. chagasi, L. braziliensis*, and *L. amazonensis* with low IC50 values (ranging from 11.86 to 17.22 μg/mL); it was also active against *T. cruzi* trypomastigotes (IC50 = 13 μg/mL). However, this compound did not show activity against the amastigote forms of *L. chagasi* and *T. cruzi*. The cytotoxicity studies using MK2 cells suggested that jacaranone is not a promising compound for treatment of leishmaniosis and Chagas disease (Morais et al., 2012).

## Compounds From *Pluchea carolinensis* Are Active Against *Leishmania* spp.

An initial screening using different extracts/fractions of *Pluchea* plants (*P. carolinensis, P. rosea* and *P. odorata*), revealed that ethanol (IC50 = 30.4 μg/mL; SI = 6) and n-hexane (IC50 = 54.5 μg/mL; SI = 4) extracts from *Pluchea carolinensis* (Jacq.) D.Dom were the most promising anti-*L. amazonensis* agent. The author also reported that the intraperitoneal administration of the ethanol extract (100 mg/kg) reduced the formation of lesions induced by *L. amazonensis* in mice (García et al., 2011).

Other work evaluated the *in vitro* and *in vivo* anti-*Leishmania* action of major phenolic constituents of *P. carolinensis* (caffeic acid, chlorogenic acid, ferulic acid, quercetin, and rosmarinic acid). All compounds inhibited promastigotes (IC50 = 0.2–0.9 μg/mL) and intracellular amastigotes (IC50 = 1.3–2.9 μg/mL). Caffeic acid (IC50 = 180.5 μg/mL), ferulic acid (IC50 = 129.03 μg/mL) and rosmarinic acid (IC50 = 93.1 μg/mL) were selected after cytotoxicity testing toward mouse peritoneal macrophages, with SI values of 11, 17, and 20, respectively. These three compounds were efficient in an experimental cutaneous leishmaniasis model induced by *L. amazonensis*. The treatment started 15 days after the infection and was done in five doses (30 mg/kg by intralesional route) each 4 days. All compounds showed *in vivo* efficacy higher than glucantime; ferulic acid showed the best active reducing the lesion size and parasite burden (Montrieux et al., 2014).

*P. carolinensis* EO also showed activity against both amastigote (IC50 = 6.2 μg/mL) and promastigote (IC50 = 24.7 μg/mL) forms of *L. amazonensis*, while cytotoxicity assay revealed a CC50 value of 28.3 μg/mL against peritoneal macrophage from BALB/c (SI = 5). The intralesional application of this EO (30 mg/kg) resulted in the reduction of parasite burden and lesion size in mice, even when compared with those animals treated with Glucantime^®^. The major component in this EO was selin-11-en-4α-ol (about 51%), however, the authors did not tested it (García et al., 2017).

## Thiophene Derivatives Isolated From *Porophyllum ruderale* Are Active Against *Leishmania* spp.

*Porophyllum ruderale* (Jacq.) Cass. is a plant used in folk medicine to treat leishmaniasis. Based on this, the inhibitory activity of the dichloromethane extract obtained from the aerial parts of *P. ruderale* and its compounds were evaluated against *L. amazonensis*. The dichloromethane extract was active for both promastigote (IC50 = 60.3 μg/mL; SI = 8.3) and amastigote (IC50 = 77.7 μg/mL; SI = 6.5 μg/mL) forms. The cytotoxicity was determined using J774G8 macrophages. The bio-guided isolation lead to the identification of two thiophene derivatives as active compounds: 5-methyl-2,2′:5′,2″-terthiophene and 5′-methyl-[5-(4-acetoxy-1-butynyl)]-2,2′-bithiophene. The compound 5-methyl-2,2′:5′,2″-terthiophene showed the best action with an IC50 value of 7.7 μg/mL (against promastigotes) and 19.0 μg/mL (against amastigotes) with SI values of 48.2 and 19.1, respectively. Meanwhile, 5′-methyl-[5-(4-acetoxy-1-butynyl)]-2,2′-bithiophene showed an IC50 and SI values of 21.3 μg/mL and 15.7 for promastigotes; and 28.7 μg/mL 11.7 for amastigotes (Takahashi et al., 2011).

In addition, Takahashi et al. (2013) provided some insights into the action of these thiophene derivatives. The authors showed that although both compounds were not able to induce damage in the parasite membrane, the 5-methyl-2,2′:5′,2″-terthiophene provoked depolarization of mitochondrial membrane potential of *L. amazonensis* promastigotes. The ultrastructural analysis confirmed this effect since mitochondria swelling were observed for promastigote and amastigote forms treated with 5-methyl-2,2′:5′,2″-terthiophene (Takahashi et al., 2013).

## Sesquiterpene Lactones From *Smallanthus sonchifolius* Are Active Against Trypanosomatids

The plant *Smallanthus sonchifolius* (Poepp.) H.Rob. has also been demonstrated as a source of sesquiterpene lactones with activity against trypanosomatids. A bio-guided assay using the epimastigote forms of *T. cruzi* led to the isolation of three active compounds from the dichloromethane extracts from *S. sonchifolius* leaves: enhydrin (IC50 = 0.84 μM), uvedalin (IC50 = 1.09 μM), and polymatin B (IC50 = 4.90 μM). Enhydrin and uvedalin were active against trypomastigotes with an IC50 of 33.4 μM and 25.0 μM, respectively. Polymatin B did not inhibit the trypomastigote form. In addition, these sesquiterpene lactones inhibited the amastigote forms with uvedalin showing the best activity (IC50 = 1.09 μM), followed by enhydrin (IC50 = 3.34 μM) and polymatin B (IC50 = 9.02 μM). Finally, the toxicity was evaluated against Vero cells, revealing that all compounds have more specificity for the amastigotes, as the SI were 16.3, 14 and 9 for polymatin B, uvedalin, and enhydrin, respectively (Frank et al., 2013).

The *in vivo* effects of uvedalin and enhydrin was evaluated in a model of *T. cruzi* infection in mice. Both compounds were administrated by intraperitoneal injections (1 mg/kg of body weight/day) on the 7th day post-infection and the treatment was performed for 5 consecutive days. The animals treated with uvedalin or enhydrin exhibited lower levels of parasitaemia, and these effects were similar to those obtained with benznidazole (positive control). Mice treated with these sesquiterpene lactones also showed higher survival ratios and reduced weight loss when compared to untreated animals (Ulloa et al., 2017).

Enhydrin, uvedalin, and polymatin B also showed anti-*Leishmania* activity when tested against *L. mexicana*. These compounds showed high leishmanicidal activity toward the promastigote form with IC50 values of 0.92 μM (enhydrin), 0.93 μM (uvedalin), and 1.04 μM (polymatin B). The action of these sesquiterpene lactones was better than the positive control, amphotericin B (IC50 = 2 μM). As seen with the anti-*T. cruzi* activity, the highest activity toward intracellular form of *L. mexicana* was observed for uvedalin (IC50 = 1.89 μM), followed for enhydrin (IC50 = 3.66 μM; Ulloa et al., 2017).

## Flavonoids From *Stevia satureifolia* Are Active Against Trypanosomatids

The dichloromethane extract from aerial parts of *Stevia satureifolia* (Lam.) Sch. Bip. var. *satureifolia* showed inhibitory action against *L. braziliensis* and *T. cruzi*. A bio-guided approach resulted in the isolation of two active flavonoids: eupatorin (IC50 = 0.2 μg/mL for amastigotes and 61.8 μg/mL for trypomastigotes) and 5-desmethylsinensetin (IC50 = 0.4 μg/mL for amastigotes and 75.1 μg/mL for trypomastigotes). 5-desmethylsinensetin showed the best activity against *L. braziliensis* promastigotes (IC50 = 37.0 μg/mL). Both compounds had low cytotoxicity toward Vero cells (CC50 > 500 μg/mL; and SI > 13.5; Beer et al., 2016).

## Sesquiterpene Lactones From *Tanacetum parthenium* Are Active Against Trypanosomatids

Two sesquiterpene lactones with activity against trypanosomatids were isolated from *Tanacetum parthenium* (L.) Sch.Bip.: guaianolide and parthenolide. Guaianolide was obtained from the hydroalcoholic extract of the aerial parts of *T. parthenium*, and it showed an IC50 value of 2.6 μg/mL toward promastigote forms of *L. amazonensis*. It was also active against the amastigote form, reducing their survival to 10% when compared to untreated cells. The cytotoxicity analysis, carried out with J774G8 cells, revealed that this compound displayed a high selectivity toward the parasite (SI = 385). The effects of guaianolide on promastigotes were associated to severe morphological alterations including changes in size, shape and number of flagellum (Da Silva et al., 2010).

Guaianolide was also effective against all forms of *T. cruzi* with IC50 values of 5.7 ± 0.7, 18.1 ± 0.8, 66.6 ± 1.3 μM for trypomastigote (SI = 16.4), epimastigote and amastigote (SI = 1.40) forms. The ultrastructural modifications induced by guaianolide involved the reduction of cell size for trypomastigotes and epimastigotes; and decrease in mitochondrial membrane potential in epimastigotes. Further, guaianolide also exhibited synergistic effect with benznidazole against the epimastigote forms and additive effects against the trypomastigote forms (Cogo et al., 2012).

Similarly, parthenolide was also isolated from the aerial parts of *T. parthenium* and exhibited activity against *L. amazonensis* (Tiuman et al., 2005) and *T. cruzi* (Izumi et al., 2008). When concerning the anti- *L. amazonensis* activity, parthenolide showed IC50 values of 0.37 μg/mL and 0.81 μg/mL toward promastigote and amastigote forms, without inducing toxic effects against J774G8 macrophages and sheep erythrocytes. The leishmanicidal activity was associated to an increase in the lysosomes size and in the exocytose in the region of the flagellar pocket (Tiuman et al., 2005). New insights on the action mechanism of parthenolide against amastigote forms of *L. amazonensis* were provided by the work of Tiuman et al. (2014). This research showed that parthenolide effect was associated with the appearance of autophagic vacuole, loss of membrane integrity, and mitochondrial dysfunction. In addition, parthenolide did not induce genotoxic effects in mice, as evaluated by micronucleus test (Tiuman et al., 2014).

In relation to anti-*T. cruzi* action, parthenolide showed an IC50 of 0.5 μg/mL against epimastigote forms and reduced the internalization of trypomastigotes forms of *T. cruzi* in LLMCK2 cells (51 and 96% when the cells were treated at 2 and 4 μg/mL, respectively). The compound also exhibited low toxicity against LLMCK2 cells with a SI of 6.4. Parthenolide induced severe alterations on the parasite, that included increase in the number of nucleus, vacuoles and reservosomes, mitochondrion swelling and the distortion of internal membranes (Izumi et al., 2008). The combinatory effects of parthenolide and benznidazole toward *T. cruzi* were also evaluated. This combination was synergistic against epimastigotes, while an additive effect was observed against trypomastigote forms (Pelizzaro-Rocha et al., 2010).

## Compounds From *Tithonia diversifolia* Are Active Against Trypanosomatids

*Tithonia diversifolia* (Hemsl.) A.Gray is a plant native of Mexico used in folk medicine that has anti-inflammatory, antimalarial, and antioxidant properties (Di Giacomo et al., 2015; Mabou Tagne et al., 2018). *T. diversifolia* dichloromethane leaf rinse extract (LRE) presents strong *in vitro* antileishmanial activity against promastigotes of *L. braziliensis* (IC50 = 1.5 μg/mL). Eight sesquiterpene lactones were isolated from leaves extracts of this plant and seven showed anti-*L. braziliensis* promastigotes properties: 1β,2α-epoxytagitinin C (IC50 = 2.2 μg/mL; SI> 22.7), tagitinin F (IC50 = 7.4 μg/mL; SI> 6.7), tagitinin A (IC50 = 7.5 μg/mL; SI> 6.6), Guaianolide 7 (IC50 = 9.0 μg/mL; SI> 5.5), tirotundin 3-O-methyl ether (IC50 = 13.7 μg/mL; SI> 3.6), tirotundin (IC50 = 8.7 μg/mL; SI = 2.9), tagitinin C (IC50 = 3.2 μg/mL; SI = 1.4). The five compounds with higher SI values were tested against amastigote forms, and the best results were found for tirotundin 3-O-methyl ether, tagitinin F, and guaianolide 7 (De Toledo et al., 2014).

## Compounds From *Vernonia* Plants Are Active Against Trypanosomatids

Plants from *Vernonia* genus are widely used in folk medicine (Toyang and Verpoorte, 2013) and some of them have been appointed as sources of bioactive compounds against trypanosomatids. For example, EOs from different parts of *Vernonia brasiliana* (L.) Druce were evaluated against trypanosomatids. Among the tested oils, the EO from flowers showed the best result toward *L. amazonensis* promastigotes, with an IC50 of 112 μg/mL and CC50 of 115 μg/mL and 391 μg/mL against Vero (SI = 1) and RAW264.7 (SI = 3) cells, respectively. Its major components were palmitic acid (8.30 %), (Z)-hex-2-en-1-ol (6.32 %), hexacosane (4.91 %), hexan-1-ol (4.23 %), and (E)-hex-2-enal (4.04 %). Regarding the action against *T. cruzi* trypomastigotes, the root EO was the most active (IC50: 70 μg/mL; SI = 3.1). The major compounds identified in this EO were modheph-2-ene (8.69%), agurjunene (9.61%), trans-caryophyllene (10.42%), β-isocomene (10.26%), and α-isocomene (15.41%) (Martins et al., 2015). Similarly, Moreira et al. (2017) studied the effect of the EO from leaves of *Vernonia polyanthes* Less. against promastigotes forms of *L. infantum*. The oil showed an IC50 of 19.4 μg/mL, while zerumbone (one of its major compound) had an IC50 of 9.0 μg/mL. The author did not report the SI value for zerumbone (Moreira et al., 2017).

Another plant from this genus with ethnopharmacological relevance is *Vernonia scorpioides* (Lam.) Pers. From this plant was extracted lupeol, that served as starting material for a semisynthetic approach in order to obtain antileishmanial and antitrypanosomal compounds. Among the derivatives, the best activity was observed for lup-20(29)-ene-diol with an IC50 of 12.4 μg/mL against *T. cruzi* amastigotes and a CC50 of 161.5 μg/mL toward THP-1 cells (SI = 12.94); this compound did not show antileishmanial action (Machado et al., 2018).

## Xanthanolides From *Xanthium* Plants Are Active Against Trypanosomatids

Xanthanolides are bicyclic sesquiterpene lactones that have a five-membered g-butyrolactone ring fused to a seven-membered carbocycle. These compounds occur in only few species, and their richest sources are plants from the genus *Xanthium* (Vasas and Hohmann, 2011). Lavault et al. (2005) examined the leishmanicidal activities of seven xanthanolides isolated from *Xanthium macrocarpum* DC. Five xanthanolides were active against *L. infantum* and *L. mexicana*, being xanthinin the most active compound with an IC50 values of 3.6 and 4.7 μg/mL toward *L. infantum* and *L. mexicana*, respectively. The other isolated compounds (Xanthinosin, Xanthatin, 4-Epiisoxanthanol, 4-Epixanthanol) exhibited IC50 values ranging from 24 μg/mL (4-Epiisoxanthanol) to 38 μg/mL (Xanthatin) against *L. infantum*, and from 35 μg/mL (4-Epixanthanol) to 44 μg/mL (Xanthinosin) toward *L. mexicana*. The authors did not report any data about the toxicity of these compounds (Lavault et al., 2005).

Bioactive xanthanolides were also obtained from *Xanthium brasilicum* Vell and tested against *T. cruzi* intracellular amastigotes *L. donovani* axenic amastigotes. Only three compounds showed anti-*T. cruzi* activity, however the low SI values (tested against L6 cells) demonstrated their small efficiency. The SI values were 1.97 for 8-Epixanthatin 1β,5β-epoxide, 1.15 for 8-Epixanthatin and 0.59 for Pungiolide A. On the other hand, all isolated xanthanolides were active against *L. donovani* and the best SI value were found for 8-Epixanthatin 1β,5β-epoxide (SI = 36.94), followed by 4,15-Dinor-1,11(13)-xanthadiene-3,5β:12,8β-diolide (SI = 14.22) and Xanthipungolide (SI > 5.84) (Nour et al., 2009).

## α-Bisabolol and α-Bisabolol-rich Essential Oil Are Active Against *Leishmania* spp.

The sesquiterpene α-bisabolol has been pointed out as a promising agent against *Leishmania* spp. (Morales-Yuste et al., 2010; Rottini et al., 2015). This compound is found in essential oils from Asteraceae plants, such as *Vanillosmopsis arborea* Barker (Colares et al., 2013), *Matricaria chamomilla* (Andrade et al., 2016), and *Matricaria recutita* L (Morales-Yuste et al., 2010; Hajaji et al., 2018). Colares et al. (2013) reported that the *V. arborea* EO and α-bisabolol were active against *L. amazonensis* with IC50 values for promastigotes of 7.35 and 4.95 μg/mL, respectively; and IC50 values for amastigotes of 12.58 and 10.70 μg/mL. These agents also provoked low cytotoxic effects in J774.G8 macrophages (SI values around 20). In addition, accumulation of electrodense lipid inclusions vesicles was induced in promastigotes treated with *V. arborea* EO and α-bisabolol (Colares et al., 2013).

In turn, *M. recutita* EO showed an IC50 value of 10.8 and 10.4 μg/mL toward *L. amazonensis* and *L. infantum* promastigotes. Following, a bio-guided fractionation of the EO constituents resulted in the identification of α-bisabolol as a major compound. α-Bisabolol showed higher IC50 values for promastigotes (16.0 and 9.5 μg/mL for *L. amazonensis* and L*. infantum*, respectively). The efficacy of α-bisabolol on amastigotes of both studied species was also assessed, and IC50 values of 5.9 μg/mL (*L. amazonensis*) and 4.8 μg/mL (*L. infantum*) were obtained. The cytotoxic evaluation of α-bisabolol was performed using J774A.1 macrophages and revealed SI values of 5.4 and 6.6 for *L. amazonensis* and *L. infantum*, respectively. The SI value for *L. amazonensis* was lower than that reported by Colares et al. (2013). The action of α-bisabolol is associated to a damage in the parasite membrane, phosphatidylserine externalization, and to a decrease in the mitochondrial membrane potential and total ATP levels (Hajaji et al., 2018). Similar results were obtained by Corpas-López et al. (2016a) that showed that α-bisabolol induced apoptosis in *L. infantum*, which is related to mitochondrial dysfunction and oxidative stress (Corpas-López et al., 2016a).

Moreover, α-bisabolol was evaluated in a murine model of visceral leishmaniasis induced by *L. infantum*. The daily oral treatment with α-bisabolol (at 50, 200, or 1,000 mg/kg doses) started 28 days after *L. infantum* infection and continued for 14 days. The best results were seen for animals treated with α-bisabolol at 200 mg/kg, where the reduction on parasite levels on spleen and liver were 71.60 and 89.22%, respectively. These results were even better than those observed for mice treated with meglumine antimoniate or the combination of meglumine antimoniate and α-bisabolol (Corpas-López et al., 2015). α-Bisabolol (in topical or oral treatment) was also shown to be effective in the treatment of cutaneous infection induced by *L. tropica* in hamsters (Corpas-López et al., 2016b). Recently, α-bisabolol was useful for the treatment of naturally acquired canine leishmaniasis. In this elegant work, the dogs received oral doses of α-bisabolol (30 mg/kg) during two series of 30 days, with 30 days of interval. The results showed that α-bisabolol-treated dogs showed lower levels of parasite load (in bone marrow, lymph node and peripheral blood) than the dogs treated with meglumine antimoniate. α-Bisabolol treatment also increased the expression of IFN-γ (Corpas-López et al., 2018).

## Other Compounds From Asteraceae Plants With Activity Against Trypanosomatids

Other compounds with activity against trypanosomatids have been isolated from the Asteraceae plants. Since the studies with these compounds only reported their isolation, they were grouped in this section. For example, the saponin Dasyscyphin C was isolated from *Eclipta prostrata* (L.) L. and showed activity against *L. major* promastigotes (IC50 = 450 μg/mL). The cytotoxicity of this saponin was not reported (Khanna et al., 2009). Similarly, Silva-Correa et al. (2018) identified an eudesman type sesquiterpen [(4αS, 5R,6R,8αR)-6-hidroxi-5,8α-dimetil-3-(1-metiletiliden) octahidronaftalen-2(1H)-ona)] as the active compound related to the leishmanicidal activity of *Tessaria integrifolia* Ruiz & Pav in *Mesocricetus auratus* infected with *Leishmania* sp. (Silva-Correa et al., 2018).

Sosa et al. (2016) evaluated the *in vitro* activity against promastigotes of *L. amazonensis* and *L. braziliensis* of seventeen sesquiterpene lactones obtained from five plants belonging to the tribe Vernonieae (*Vernonanthura pinguis* (*Griseb*.) H.Rob., *Vernonanthura nebularum* (Cabrera) H.Rob., *Eirmocephala megaphylla* (Hieron.) H.Rob., *Centratherum punctatum* subsp. *punctatum* and *Elephantopus mollis* Kunth). These sesquiterpene lactones were from six structural groups: hirsutinolides, glaucolides, germacranolides, isogoyazensolides, goyazensolide, and germacradiendiolides. The authors reported that all compounds were able to inhibit the promastigotes forms of *L. amazonensis* and *L. braziliensis*, and these results confirm the leishmanicidal potential of sesquiterpene lactones (as seen for several plants discussed in this review; Barrera et al., 2013). Among the most active compounds were isodeoxyelephantopin, deoxyelephantopin, centratherin. The authors did not report any information about the toxicity of these compounds (Sosa et al., 2016).

Two bioactive germacranolides were also found in *Neurolaena lobata* (L.) R.Br. ex Cass. named Neurolenin B and Nerolenin C/D. They presented inhibitory activities toward *L. mexicana* promastigotes (IC50 values of 3.4 and 5.5 μg/mL, respectively), *L. braziliensis* promastigotes (IC50 values of 98.5 and 188.6 μg/mL, respectively) and epimastigotes (IC50 values of 6.3 and 11.7 μg/mL, respectively) and trypomastigotes forms of *T. cruzi* (4.9 and 6.1 μg/mL, respectively; Berger et al., 2001). Similarly, sesquiterpene lactones were isolated from *Pseudelephantopus spiralis* (Less.) Cronquist with activity against amastigotes forms of *L. amazonensis*: 8,13-diacetyl-piptocarphol (IC50 = 0.2 μM), 8-acetyl-13-O-ethyl-piptocarphol (IC50 = 0.37 μM) and ursolic acid (IC50 = 0.99 μM; Odonne et al., 2011). In addition, three hirsutinolide-type sesquiterpenoids [diacetylpiptocarphol; piptocarphins A; (1S(^*^),4R(^*^),8S(^*^),10R(^*^))-1,4-epoxy-13-ethoxy-1,8,10-trihydroxygermacra-5E,7(11)-dien-6,12-olide] with activity against promastigotes and axenic amastigotes of *L. infantum* were isolated from *P. spiralis*, however these compounds were found to be cytotoxic against VERO cells (Girardi et al., 2015).

Finally, it is important to highlight that those studies without data on the isolation/chemical composition of the extracts or their effects *in vivo* were not included in this manuscript. Examples of these studies are: *Calendula officinalis* L. against *L. major* promastigotes and amastigotes (Nikmehr et al., 2014); *Echinacea purpurea* (L.) Moench against *L. donovani* promastigotes (Canlas et al., 2010) and *L. major* in *in vivo* infection (Sarkari et al., 2017); *Echinops kebericho* Mesfin toward *L. aethiopica* and *L. donovani* promastigotes and amastigotes (Tariku et al., 2011); *Gochnatia pulchra* Cabrera against *L. amazonensis* (Lucarini et al., 2012, 2016); *Waldheimia tomentosa* (Decne.) Regel against *L. major* promastigotes and amastigotes (Bhatnagar et al., 2017); and *Pulicaria gnaphalodes* (Vent.) Boiss. toward *L. major* (Asghari et al., 2014).

## Conclusion

Taken together, all these studies show that Asteraceae plants are interesting sources of compounds with inhibitory activity toward trypanosomatids. These compounds have the potential to improve the development of new effective agents against these neglected protozoan diseases. It is important to note that several of these compounds need to be evaluated with *in vivo* models. Furthermore, these papers provided scientific bases for the use of several plants with ethnopharmacological relevance in different countries.

## Author Contributions

RM and RS performed data collection and writing of the manuscript with support from LdS, TH, and AA. TH and MB contributed with the final version of the manuscript and with the important intellectual content of the study. LdS conceived the present study and design and implementation of the research and critical review of the manuscript regarding the important intellectual content of the study. AA contributed with the design of the research, data collection and supervised the work of RM and RS.

### Conflict of Interest Statement

The authors declare that the research was conducted in the absence of any commercial or financial relationships that could be construed as a potential conflict of interest.
